# Participation of the Serotonergic System and Brain-Derived Neurotrophic Factor in the Antidepressant-like Effect of Flavonoids

**DOI:** 10.3390/ijms231810896

**Published:** 2022-09-17

**Authors:** León Jesús German-Ponciano, Gilberto Uriel Rosas-Sánchez, Jonathan Cueto-Escobedo, Rafael Fernández-Demeneghi, Gabriel Guillén-Ruiz, César Soria-Fregozo, Emma Virginia Herrera-Huerta, Juan Francisco Rodríguez-Landa

**Affiliations:** 1 Instituto de Neuroetología, Universidad Veracruzana, Xalapa 91190, Mexico; 2 Departamento de Investigación Clínica y Traslacional Instituto de Ciencias de la Salud, Universidad Veracruzana, Xalapa 91190, Mexico; 3 Facultad de Nutrición, Universidad Veracruzana, Xalapa 91017, Mexico; 4 Programa de Investigadoras e Investigadores por México CONACyT-Instituto de Neuroetología, Universidad Veracruzana, Xalapa 91190, Mexico; 5 Centro Universitario de Los Lagos, Universidad de Guadalajara, Lagos de Moreno 47460, Mexico; 6 Facultad de Ciencias Químicas, Universidad Veracruzana, Orizaba 94340, Mexico; 7 Laboratorio de Neurofarmacología, Instituto de Neuroetología, Universidad Veracruzana, Xalapa 91190, Mexico

**Keywords:** depression, flavonoid, serotonin, BDNF, polyphenol

## Abstract

Depressive disorders are among the most disabling diseases experienced around the world, and their incidence has significantly increased over the last few decades due to multiple environmental, social, and biological factors. The search for new pharmacological alternatives to treat depression is a global priority. In preclinical research, molecules obtained from plants, such as flavonoids, have shown promising antidepressant-like properties through several mechanisms of action that have not been fully elucidated, including crossing of the blood brain barrier (BBB). This review will focus on discussing the main findings related to the participation of the serotonergic system and brain-derived neurotrophic factor (BDNF) on the antidepressant-like effect of some flavonoids reported by behavioral, neurochemical, and molecular studies. In this sense, evidence shows that depressive individuals have low levels of serotonin and BDNF, while flavonoids can reverse it. Finally, the elucidation of the mechanism used by flavonoids to modulate serotonin and BDNF will contribute to our understanding of the neurobiological bases underlying the antidepressant-like effects produced by these natural compounds.

## 1. Introduction

The COVID-19 pandemic has had a colossal negative impact on mental health around the world [1]. Loss of family and loved ones, fear of infection, isolation, job loss, schooling from home, and financial concerns are powerful stressors that have been witnessed during this pandemic, contributing to the development of feelings of loneliness and anger, and increasing the prevalence of mental disorders such as depression. According to the World Health Organization, the worldwide prevalence of depression symptoms exponentially increased by 25% during the last two years [2]. Depression is one of the main comorbid disorders present in several disabling conditions such as cardiovascular diseases, diabetes, rheumatoid arthritis, and cancer, among others [3], and it represents the main economic burden of all mental illnesses [4]. Therefore, this psychiatric disorder is considered one of the most important subjects of concern in healthcare around the world [2]. From this perspective, there is a clear urgent need to develop methods that aim to care for people’s mental health. The search for new drugs for the treatment of depression has become even more urgent during this pandemic [5,6].

There are several pharmacological treatments to relieve the symptoms of depression, which include different drugs such as selective serotonin reuptake inhibitors (SSRIs), dual antidepressants (serotonin and noradrenalin reuptake inhibitors), tricyclic antidepressants, and monoamine oxidase inhibitors (MAOIs), which increase the levels of monoamines such as dopamine (DA), noradrenaline (NE), and particularly serotonin (5-HT) [7,8]. 

Notably, it has been shown that the onset of the therapeutic effects of antidepressants is mediated by long-term neuroplasticity processes [9]. Specifically, the increase in neurotrophins such as brain-derived neurotrophic factor (BDNF) is associated with the improvement of depression symptoms in patients treated with antidepressant drugs [9,10]. However, only 50%–70% of depressed patients experience a decrease of at least 50% in symptom severity, of which only half achieve full remission [11]. In addition, the long period of latency until the manifestation of therapeutic effects [12] and experiencing side effects (i.e., sexual dysfunction, insomnia, anxiety, dry mouth, among others) [13,14] are considered as the main reasons why patients withdraw from treatment. Because of this, research conducted at the preclinical level has focused on finding new pharmacological alternatives for the treatment of depression [5,6].

Natural compounds with potential therapeutic application in neuropharmacology have gained interest in clinical and preclinical research [15]. Particularly, flavonoids have demonstrated neuroprotective properties, exerting antidepressant effects both in depressed patients and validated animal models [16,17]; however, the specific mechanism of action of flavonoids remains unclear. Thus, this review focuses on recapitulating and analyzing the main preclinical findings on the participation of the serotonergic system and BDNF in the mechanism of action responsible for the antidepressant-like effect of flavonoids. The above is important considering the link between the serotonergic system and BDNF in the neuroplasticity involved in the appearance of the therapeutic effect of antidepressant drugs. A narrative review of specialized scientific articles published in indexed scientific journals was conducted following a search for relevant articles in prestigious databases (PubMed, Scopus, Google Scholar) using the following keywords: “flavonoid, BDNF, animal model, depression, phytochemical, and 5-HT”. This review has the objective of expanding the knowledge on the neurobiological mechanisms of the antidepressant-like effect of flavonoids, particularly those related to 5-HT and BDNF changes. 

## 2. Serotonergic System and BDNF Involved in Depression Etiology and in Response to Antidepressants

Depression is a disabling psychiatric disorder that is characterized by a loss of interest in performing most daily activities, concentration problems, feelings of guilt and despair, suicidal thoughts, appetite and sleep changes, as well as low energy [2]. Depressed people are more vulnerable to developing additional chronic diseases; moreover, considering the combined disability from depression plus that produced by chronic diseases, depression can be considered as one of the most expensive medical conditions worldwide, with approximately 280 million people living with this condition [18].

Despite great progress in neurosciences and psychiatry, the etiology of depression has not been fully dilucidated [19]. However, several new theories have arisen to complement explanations on the etiology of depression [20], such as the neurotrophic hypothesis of depression, which is one of the most recently studied. This theory proposes that neuroplasticity is a key factor in depression and in the therapeutic effects of antidepressant drugs [21]. It is supported by the evidence that depressed patients present low levels of neurotrophins (i.e., BDNF, and nerve growth factor = NGF) [22,23,24]. In addition, chronic treatment based on antidepressant drugs has been shown to reestablish the levels of these neurotrophins [24,25,26], promoting neuroplasticity in brain structures involved in mood and emotional regulation such as the prefrontal cortex (PFC) and hippocampus (HP), among others [9,27,28].

Interestingly, 5-HT and BDNF represent two of the most important systems involved in neuroplasticity [29,30]. Of these, 5-HT is perhaps the most studied neurotransmitter associated with the etiology of depression, since it was initially proposed that low levels of 5-HT are related with the risk of depression [31], while levels of this monoamine increase following antidepressant treatment [32]. However, it is a first step in the establishment of depression and the therapeutic effect of antidepressant drugs, because multiple neuroplasticity events are occurring in parallel. On the other hand, BDNF is a neurotrophin that has been linked to depression in addition to having essential functions in neuroplasticity, neuronal survival, and neurogenesis [33]. 5-HT plays an essential role in the mechanism of action of antidepressant drugs (i.e., SSRIs, MAOIs, tricyclics, and duals) due to the rapid and transient increase in 5-HT bioavailability in the synaptic space [34]. Hence, since pharmacological treatment only exerts therapeutic effects after several weeks, it has been assumed that the long-term improvement of serotonergic neurotransmission can promote greater expression of neuropeptides associated with neuroplasticity, such as BDNF [29], which is known to be modulated by different classes of antidepressant drugs, particularly SSRIs [35]. In this sense, a recent study examined 51 depressed patients that presented low levels of BDNF in serum compared to the control group. However, after 8 weeks of fluoxetine, escitalopram, and paroxetine administration, depressed patients had significantly increased serum BDNF levels, which reached values similar to those of the control group. These effects were negatively correlated with scores based on the Hamilton Rating Scale for Depression (HRSD) [36]; that is to say, when BDNF levels increase, the HRSD score decreases. This implicates BDNF in the therapeutic effects of antidepressant drugs. 

Preclinical research also provides support implicating 5-HT and BDNF in the antidepressant effects [37,38,39]. Several studies have focused on exploring the interaction between both systems and its effects in terms of depressive-like behavior in animal models [40,41,42,43]. In vitro studies using B-lymphoblast cell culture have shown that exposure to 2% BDNF medium promotes a decrease in 5-HT reuptake [44], which is associated with the increase in 5-HT viability, as reflected in the reduction of depressive-like behavior in animal models [45]. Similarly, in a study carried out in Slc6a41Hubr rats with knockout of the 5-HT transporter (SERT−/−), a decrease in the BDNF levels was detected, which was associated with a reduction in sucrose intake (anhedonia) and severe depression-like behavior in the forced swim test (FST). Contrarily, BDNF overexpression was associated with increased sucrose intake and a decrease in depression-like behavior [43]. Similar effects were produced by 5-HT depletion, via the administration of p-chlorophenyl-alanine (10 mg/kg, s.c.), which promoted a decrease in BDNF in the HP and PFC of rats, and high levels of anxiety- and depressive-like behaviors [46]. 

In summary, preclinical and clinical evidence shows that 5-HT and BDNF have a bidirectional relationship, where the modification of one may affect the other, which can have a direct impact in the establishment of both depression symptoms and the therapeutic effects of antidepressants. This places 5-HT and BDNF as the main targets of new molecules for depression disorders.

## 3. Serotonergic System

The serotonergic neurons are in the midbrain (brain stem), specifically in the raphe nucleus, where axons innervate several brain structures, particularly those of the brain cortex, limbic system, basal ganglia, and spinal cord [47,48,49]. These neurons synthesize 5-HT from the amino acid tryptophan, which is converted into 5-hydroxytryptophan (5HTP) by the enzyme tryptophan hydrolase type 2 (TPH2). 5-HTP is converted to 5-HT by the action of the enzyme aromatic amino acid decarboxylase [50]. The action of 5-HT is mediated by seven families of receptors (5-HT_1_–5-HT_7_), which are subdivided into 14 subtypes, all of which are coupled to G protein, except for the 5-HT_3_ receptor, which is a ligand-gated ion channel [51]. In this sense, the 5-HT receptors are expressed throughout the peripheric and central nervous system (CNS), particularly in areas involved in the neurobiology of depression. 

5-HT participates in the regulation of the sleep–wake cycle, aggressiveness, motivation, sexual behavior, and neuroendocrine activity through the hypothalamic–pituitary–adrenal axis, which is the main stress response system. Dysregulation of the serotonergic system is involved in the physiopathology of depression, while pharmacological treatment with antidepressant drugs (i.e., SSRIs, tricyclics, MAOIs) reduces symptoms of depression [52,53]. Thus, the decrease in 5-HT levels and serotonergic projections to brain areas involved in the regulation of mood, as well as an increase in the autoinhibition of 5-HT and a deficient antidepressant response, are involved in the establishment of depression [54,55].

In the 1960s, the monoaminergic hypothesis was proposed, whereby a decrease in several neurotransmitters such as 5-HT, DA, and NE in the CNS is responsible for the development of depressive symptoms [56,57,58]. This hypothesis is supported by the mechanism of action of antidepressant drugs, which increase monoamine levels and alleviate depressive symptoms [59,60]. Nevertheless, this hypothesis does not explain the delay in the therapeutic effect of antidepressant drugs. Since inhibition of the reuptake or metabolism of monoamines occurs within a matter of hours, it is not directly related to any therapeutic effects that occur after several weeks of treatment [61]. Attempts have been made to explain this discrepancy between the pharmacological action and therapeutic effect of antidepressants, and it was shown that the sensitivity of 5-HT receptors plays a key role in said mechanisms. In this sense, long-term receptor desensitization promotes an increase in the firing rate of 5-HT neurons [34,62], which is associated with those slow adaptive changes induced in the 5-HT receptors that could explain the long latency to the antidepressant effect [34,63].

Additionally, stimulation of the 5-HT_1A_ and 5-HT_2A_ receptors produces changes in downstream signaling, regulating gene expression via the activation of diverse genes that in turn regulate the transcription of messenger ribonucleic acid (mRNA). The protein cAMP response element binding protein (CREB) is one of these transcription factors involved in learning and memory, synthesis of neurotrophins, circadian cycles, neurogenesis, and the pathophysiology of psychiatric and neurodegenerative disorders [64]. In this sense, the activation of CREB, through the chronic administration of antidepressant drugs, has been strongly linked to the promotion of neuroplasticity processes in which neurotrophic factors such as BDNF play a fundamental role.

## 4. Brain-Derived Neurotrophic Factor

BDNF is a peptide of the neurotrophin family encoded by the *BDNF* gene, which plays an important role in the modulation of neuroplastic processes that underly learning, memory, and behavior [65]. This protein is essential for neurodevelopment, and it acts on neurogenesis, maturation, and differentiation of neurotransmission systems such as the brain stress and reward and motivation systems, which are related to mental disorders [66,67]. This neurotrophin is located in several brain structures as the HP and PFC, where its levels are high [68]. 

BDNF and its tropomyosin receptor kinase B (TrkB) are associated with the development of mood disorders and establishment of therapeutic effects of antidepressant drugs [69]. In fact, Duman et al. was the first to propose the connection between BDNF, depression, and the antidepressant action [54]. In this sense, Nibuya et al. (1995) reported that BDNF levels increased in the HP and PFC of Sprague-Dawley male rats 18 days after electroconvulsive therapy [70]. Later, it was verified that pharmacological and nonpharmacological antidepressant therapies (i.e., drugs, cognitive behavioral therapies) can also increase the concentrations of BDNF in different brain areas [35]. 

The above is supported by subsequent studies in which both BDNF mRNA and protein levels were decreased in the brain of postmortem depressive patients [71], particularly in the HP [72] and amygdala [73]. The same effect was found in suicide victims compared with healthy subjects [74]. Furthermore, DNA methylation of BDNF gene promoters is increased in the peripheral blood mononuclear cells of depressed patients [75] and samples from suicide patients [76], which is consistent with a reported reduction in BDNF expression in depressed patients [71]. 

The reduction of BDNF levels is not specific to depression, and similar reductions have been observed in other neuropsychiatric disorders, such as schizophrenia and dementia [77,78]. In preclinical research, stressors are often used to trigger depression-related behavior. Stress decreases BDNF expression in several brain regions such as HP [79,80] and increases BDNF expression in other brain regions such as basolateral amygdala [81] depending on the type and duration of the stressor [82]. However, the high inter- and intra-individual variation in serum BDNF levels prevents the generalized use of BDNF as a biomarker of depression [83].

In addition to BDNF, its receptor TrkB is implicated in depression disorders and the therapeutic effects of antidepressant drugs [84]. For example, the TrkB protein and its mRNA are decreased in postmortem brain samples from depressed patients [70,85]. Moreover, it was found that activated phosphorylated forms of TrkB are decreased in brain samples from depressed patients [86]. In accordance, the increase in TrkB activation is associated with a decrease in depressive-like behavior in mouse models [87]. In this sense, BDNF depletion did not affect the antidepressant response as observed in behavioral models, though the loss of TrkB resulted in an attenuated response to antidepressant drugs [88], which reveals a critical role for the TrkB receptor in conventional antidepressant action that is independent of BDNF signaling. Moreover, TrkB overexpression can produce antidepressant-like effects in male C57BL/6 mice subjected to FST [89]. 

Recently, it was shown that antidepressant drugs such as fluoxetine, imipramine, and ketamine, can bind directly to the TrkB receptor [90]. This affinity is lower than that for the 5-HT transporter; however, antidepressants accumulate in the brain, and the concentrations necessary for TrkB binding are achieved after several weeks of treatment [91]. These findings have promoted the development of a potential hypothesis, which proposes that the primary site of action of antidepressant drugs is direct binding to TrkB receptor rather than monoamine transporters [82].

The aforementioned evidence supports the crucial role of BDNF and its TrkB receptor in depression, since signaling dysregulation of these two components is associated with reduced neuroplasticity and the development of depression symptoms. However, it is important to note that BDNF is not only one of the main targets of conventional antidepressant drugs but also represents a cell target for new molecules with potential antidepressant effects.

## 5. Searching for Alternatives to Antidepressant Drugs

Despite the great advances in pharmacological research on antidepressant drugs, even during the latency period with new treatments patients continue suffering from depressive symptoms and some even drop out of treatment [92]. In addition, some patients have an increased risk of suicide during the first week of pharmacological treatment [93]. These characteristics drive the search for new active compounds with faster effects such as probiotics [94] or ketamine, whose effects are related to rapid molecular neuroplasticity; however, their clinical use is unfortunately limited by its poor safety and development of pharmacological tolerability [95,96]. As a consequence, the identification, evaluation, and development of new antidepressant substances with improved efficacy and apparently fewer side effects has become the main objective of numerous studies [16]. 

In this sense, the study of phytochemical compounds, such as flavonoids, is a growing field in neuropharmacology research [97,98], especially due to their impact on the CNS, including their potential antidepressant-like effects [16,99,100].

## 6. General Information of Flavonoids

Flavonoids are a group of polyphenolic compounds produced by plants as secondary metabolites. They act as signal compounds, attracting pollinators or animals for seed dispersion and protecting plants from oxidants and ultraviolet radiation [101]. These phenolic compounds are widely found in fruits and vegetables [102,103]. Those termed as “dietary flavonoids” constitute an important component of the normal human diet [104] and have been implicated in conferring a large range of health benefits arising from their bioactive properties, such as anti-inflammatory, anticancer, anti-aging, cardio protective, immunomodulatory, antidiabetic, antibacterial, antiparasitic, antiviral, and neuroprotective effects [104,105,106].

Flavonoids have been considered as a nutraceutical product in recent research [107]. Their antioxidant activity has been extensively studied in vitro, which can prevent damage caused by free radicals through scavenging of reactive oxygen species (ROS), upregulation of intracellular production of antioxidant enzymes, inhibition of free radical generating enzymes (i.e., xanthine oxidase, lipoxygenase, protein kinase) [108], and reduction of α-tocopherol radicals [109]. Moreover, it has been suggested that the presence of various functional groups (i.e., the hydroxyl group –OH), as well as their number and location in their chemical structure, could be responsible for these antioxidant properties [110].

Flavonoids are frequently found as glycosylated or esterified forms. These share a basic 15-carbon skeleton consisting of single-bond C3 and C6 rings, namely rings A and B linked by a third carbon ring [111]. In fact, it has been proposed that catechol (o-dihydroxy) or pyrogallol (trihydroxy) groups attached to ring B are essential for its antioxidant activity, since they require less energy for their dissociation [112] and are therefore more capable of scavenging free radicals directly by donating a hydrogen atom [113]. Some studies have reported that daily intake of flavonoids reduces the risk of diverse diseases, including cancer, cardiovascular disease, and neurodegenerative and psychiatric disorders [114], and it has even been considered that the antiviral properties of flavonoids could also be applied in treatment in the context of the current COVID-19 pandemic [115].

However, even though these compounds have been recognized as powerful antioxidants, little has been mentioned about their prooxidant action [113], which has been proposed to be directly proportional to the number of hydroxy groups, since mono- and dihydroxy compounds show no detectable prooxidant effects, while poly-hydroxy flavonoids present strong prooxidant activity [116]. It has also been observed that the prooxidant activity of these secondary metabolites is concentration dependent [117]. For example, in a study conducted on rat liver cells, quercetin was found to inhibit lipid peroxidation at micromolar concentrations (≤1.5 μM); however, at 100 μM, it enhanced the formation of hydroxy radicals [118].

Based on in vivo studies, there is no clear evidence regarding the prooxidant properties of flavonoids, since there are no studies available on whether these compounds have the same actions in the stomach, intestine, or colon of humans [119], which is mainly related to their poor absorption in the digestive tract [120]. However, it has been proposed that even if there are slight prooxidant properties in the human body, these would be beneficial, since flavonoids would possibly promote a certain degree of oxidative stress and, therefore, the increased levels of defense and antioxidant enzymes would lead to an overall boost to cytoprotection [121]. It is important to highlight that the reported prooxidant properties of flavonoids could be also related to potential side effects in the long-long term, considering that the prooxidant properties of several synthetic and natural products may result in the deterioration of cellular membranes and increase in the nonselective filtration of substances and, thus, negatively impact on cellular function [122]. In this way, it is necessary that, in addition to studying their beneficial effects on health, specific studies also evaluate the potential side effects of flavonoids to identify possible limitations for excluding them from consideration as therapeutics of several illness in humans.

There are different types of flavonoids, and they are classified depending on their chemical structure, degree of unsaturation, and oxidation of carbon rings. The subclasses include flavonols, flavones, flavanones, flavanols, isoflavones, and anthocyanidins [123,124], and they can be associated with different colors in the diet and can be obtained from different vegetal sources (Table 1).

### 6.1. Flavonols

Flavonols have a 3-hydroxyflavone backbone that consists of hydroxyl groups at different positions that can be used to synthesize many compounds with a different activity [125]. Kaempferol, quercetin, myricetin, and fisetin are some of the most studied flavonols due to their antioxidant properties [124]. Dietary flavonols are bioavailable molecules with human health benefits; for example, quercetin inhibits ROS-mediated hepatocarcinogenesis by upregulating enzymatic (catalase (CAT), superoxide dismutase (SOD), glutathione peroxidase, paraoxonase) and nonenzymatic (total glutathione) antioxidant defense systems [126]. In addition, flavonols are associated with antidepressant and anxiolytic activities, possibly after increasing 5-HT and decreasing 5-hydroxyindoleacetic acid (5-HIAA) in the brain [127].

### 6.2. Flavones

Flavones are one of the largest and important subgroups of flavonoids. They are widely distributed as glucosides in leaves, flowers, and fruits. Among the main flavones, the following stand out due to their actions on CNS: luteolin, apigenin, tangeretin, and chrysin [128]. In this sense, the biological effects of apigenin are related to gene transcription, protein expression, and enzyme activity levels, and reducing antioxidant enzyme loss in streptozotocin-treated cells [129]. In addition, there is evidence showing that chrysin exerts anxiolytic- and antidepressant-like effects, which have been associated with the GABA_A_ receptor modulation and the increase of BDNF in the brain, as well as the regulation of 5-HT receptors at pre- and post-synaptic levels [130,131,132].

### 6.3. Flavanones

Flavanones are present in all citrus fruits such as oranges, lemons, and grapes. Hesperitin, naringenin, and eriodictyol are examples of this class of flavonoids [133]. Flavanones can provide phenolic hydrogen, thereby functioning as an effective antioxidant [134]. Furthermore, flavanones can favor the production of glial cell line-derived neurotrophic factor against Parkinson’s disease through anti-inflammatory effects, preventing neurodegeneration [135].

### 6.4. Flavanols

Flavanols are generally present in two forms: proanthocyanidins and catechins. These compounds are found abundantly in bananas, apples, blueberries, peaches, pears, and apricot, among others [136]. The long-term consumption of flavanol-rich foods facilitates the improvement of endothelial function and prevents the development of cardiovascular diseases [137]; moreover, anti-inflammatory and vasodilatory activities have been identified in the flavanols [138], and they can also induce positive effects on cognitive processes, including in relation to attention, working memory, and processing speed [139].

### 6.5. Isoflavones

Isoflavones are predominantly found in soybeans and other leguminous plants [140]. These flavonoids are a subgroup in which the B ring is attached to position three of the C ring. They have structural similarities to estrogens, such as estradiol, and for this reason they are also called phytoestrogens (i.e., daidzein and genistein) [114,141]. In this sense, isoflavones have gained popularity as an alternative treatment for menopausal symptoms in women who are unwilling or cannot take hormone replacement therapy [142]. In addition, isoflavones can improve cognitive function and relieve depressive symptoms [143].

### 6.6. Chalcones

Chalcones are characterized by the absence of the C ring of the basic flavonoid skeleton structure. Hence, they can also be referred to as open-chain flavonoids. Major examples of chalcones include phloridzin, arbutin, phloretin, and chalconaringenin [124]. They exhibit antioxidant, antibacterial, anthelmintic, antiulcer, antiviral, antiprotozoal, and anticancer effects [144]. Recent work evidenced antidepressant activity on the FST and tail suspension test (TST) of a new chalcone compound denominated DHIPC (2,4-dichloro-2′-hydroxyl-4′,6′-diisoprenyloxychalcone) as capable of increasing the concentrations of 5-HT and NE, and it increases 5-HIAA contents in the HP, hypothalamus, and cortex in the brain [145].

### 6.7. Anthocyanidins

Anthocyanidins are a class of water-soluble flavonoids and natural pH indicators [146]. They are commonly present in higher plants and are mainly responsible for the blue, purple, and red colors of fruits such as berries, grapes, and certain tropical fruits in addition to vegetables, roots, and cereals. In addition, anthocyanidins help to attract insect pollinators. The six most common types are cyanidin, pelargonidin, delphinidin, peonidin, petunidin, and malvidin [147]. Anthocyanidins have diverse biological activities such as antioxidant, antiproliferative, and anti-inflammatory properties [133]. Currently, the scientific literature indicates their potential effects as neuroprotectors on diseases such as anxiety, depression, Alzheimer’s, and Parkinson’s diseases [148].

Finally, flavonoids possess several biological properties. Intriguingly, the importance of flavonoids in different neurological disorders has gained increasing attention due to their actions on the CNS and regulation of emotional and mood processes associated with neurochemical and neuroplastic changes, such as antidepressant drugs [16]. In addition, at the clinical level, recent meta-analysis studies have demonstrated the potential antidepressant effects of flavonoids. However, more knowledge about its clinical application is also necessary, mainly about the optimal doses, duration of treatment, and amount of intake [149]. Despite this, flavonoids could, in the median and long term, represent one of the main pharmacological options for the development of antidepressant drugs.

## 7. Pharmacokinetics of Flavonoids and Their Entry into the CNS

Most flavonoids are present in food in their O-glycoside form, with glucose being the most common β-linked residue, but glucoramnose, galactose, arabinose, and rhamnose are also present [150]. Once they are ingested and before entering the general circulation, these glycosides can undergo deglycosylation (hydrolysis), which takes place in either the small or large intestines depending on the type of sugar [104]. This process is carried out by two β-glucosidase enzymes: lactase-phlorizin hydrolase, which hydrolyzes lactose, glucose, and galactose, and cytosolic β-glycosidase, which has specificity dependent on the aglycone moiety [151]. The next step is the passive diffusion of the flavonoid aglycones through epithelial cells [152]. In this sense, isoflavones are the most efficiently absorbed, while flavanols and flavanones are intermediately absorbed, and proanthocyanins and anthocyanins are poorly absorbed [153]. 

After absorption, flavonoids are transported to the liver for further metabolism through different conjugation reactions such as O-methylation, sulfation, and glucuronidation. Due to flavonoids having a high conjugation capacity, their concentration in plasma is generally low [150]. These metabolites can also undergo oxidative metabolism mediated by cytochrome P450 enzymes. Likewise, metabolism can be carried out through bacteria in the colon, which hydrolyzes the parent, and in the upper part of the intestine unmetabolized flavonoids as well as their glucuronides and sulfates can be found. Some research has reported that conjugation reactions with glucuronic acid and/or sulfate are the most common for flavonoids. Finally, because of the metabolism of flavonoids, more hydrophilic compounds are obtained and hence eliminated through different routes. In the case of flavonoids, elimination in the bile is quantitatively the most important elimination route [104].

On the other hand, despite some research showing that diets rich in flavonoids have various therapeutic effects both at the systemic level and in the CNS [154,155,156,157], most studies have reported the presence of these compounds and their metabolites at the peripheral level, but little has been explored with respect to their bioavailability in the brain and the mechanisms that facilitate their transport through the blood–brain barrier (BBB) [158].

Epicatechin (a flavanol found mostly in cocoa and green tea) and its methylated form (3′-O-methyl epicatechin) were found in the brains of rats after (1, 5, and 10 days) its oral administration (100 mg/kg body weight/d) [159]. The capacity of epicatechin and its metabolite to cross the BBB in an in vitro model hCMEC/D3c cell culture has also been evaluated. Both were found to cross the BBB in a time-dependent manner (at 3 and 18 h), although with higher efficiency for the methylated metabolite. This suggests that the transport process involved is likely passive diffusion, since methylated molecules are more lipophilic than unconjugated epicatechin and, therefore, more easily cross the BBB [160]. 

Similarly, quercetin and its metabolite (3-O-glucuronyl-quercetin; 50 mg/kg body wt; p.o.) were found in rat brain tissue in a capillary endothelial cell line [161]; its transportation through the BBB was also evaluated. In this sense, it was found that quercetin and its glucuronidated form crossed the BBB (a model cell line hCMEC/D3), increasing its concentration as time passed (over 1, 3, and 18 h). However, its metabolite showed a faster rate [160].

Interestingly, in the case of anthocyanins, these compounds have only been identified intact or glycosylated (unconjugated) in the CNS [162,163]. Three anthocyanins were evaluated: delphinidin-3-O-glucoside (Dp-3-gl), cyanidin-3-O-glucoside (Cy-3-gl), and malvidin-3-O-glucoside (Mv-3-gl), and all crossed hCMEC/D3 cells in a time-dependent manner (over 1, 3, and 18 h) but showed different efficiencies associated with their hydrophilicity. Dp-3-gl is the most hydrophilic and, therefore, least efficient of the three derivatives, which suggests the influence in which the polarity of anthocyanins plays in their transport through the BBB [160]. In addition, the neuroprotective effects of flavonoids could possibly be mainly exerted by their conjugated metabolites, considering that a mixture of different conjugated quercetin metabolites was shown to exert more effective antihypertensive effects than the isolated molecule [164]. 

## 8. Participation of Serotonergic System in the Antidepressant-like Effect of Flavonoids

Diverse preclinical studies have evaluated the effect of flavonoids in promoting the development of new alternatives for treating depression [16]. In this sense, the antidepressant-like effect produced by flavonoids has been demonstrated using animal models of depression such as the FST, TST, or sucrose water consumption test [165], among others. These effects are associated with the modulation of several neurotransmission systems such as noradrenergic, dopaminergic, and serotonergic [17,130]. Table 2 summarizes the findings regarding the antidepressant potential of some flavonoids that exert their action through the serotonergic system, which has been extensively related to the etiology of depression and the mechanism of action of antidepressant drugs [130,166,167].

**Table 2:** Flavonoids with antidepressant-like effects and their action on the serotonergic system.

Flavonoid	Experimental Subjects	Treatment	Behavioral Effect	Effect on Serotonergic System	Reference
Astilbin (taxifolin-3-O-rhamnoside)	Adult male C57BL/6J mice	10, 20, and 40 mg/kg (i.p.) for 21 days	↓ TTI in FST and TST ↑ Sucrose intake	↑ 5-HT in frontal cortex	[168]
Hesperidin (3,5,7-trihydroxyflavanone-7-rhamnoglucoside)	Male adult Swiss mice	0.1, 0.3, and 1 mg/kg (i.p.) S.D. 30 min before behavioral test	↓ TTI in FST and TST	Pretreatment with pCPA (100 mg/kg, i.p.) prevents antidepressant-like effect	[169]
	Adult male Wistar rats with hyperglycemia induced by streptozotocin	25, 50, and 100 mg/kg (p.o.) for 21 days	↓ TTI in FST	↑ Brain levels of 5-HT	[170]
	Male Swiss Albino mice	1 mg/kg (i.p.) for 14 days	↓ TTI in FST and TST	↑ 5-HT in HP and cerebral cortex	[171]
	Old male Sprague-Dawley rats	20, 50, and 100 mg/kg (i.p.) for 14 days	↑ Sucrose intake ↓ TTI in FST	↑ 5-HT in HP, PFC, and amygdala	[172]
Rutin (quercetin-3-*O*-rhamnosylglucoside)	Male Swiss mice	0.01, 0.1, 0.3, 1, 3, and 10 mg/kg (p.o.) 60 min before the behavioral test	↓ TTI in FST	Pretreatment with pCPA (100 mg/kg, i.p.) prevents antidepressant-like effect	[173]
	Five weeks old male Sprague Dawley rats	225 mg/kg (p.o.) for 28 days	↓ TTI ↑ Swimming time in FST	↑ 5-HT in frontal cortex, HP, striatum, and amygdala	[174]
Icariin (7-(β-D-Glucopyranosyloxy)-5-hydroxy-4′-methoxy-8-(3-methylbut-2-en-1-yl)-3-(α-L-rhamnopyranosyloxy) flavone)	Adult male Wistar rats	30 and 60 mg/kg (p.o.) for 5 weeks	↑ Sucrose intake	↑ 5-HT_1A_ mRNA levels in HP and frontal cortex	[175]
Orientin (luteolin-8-C-glucoside)	Adult male Kunming mice	20 and 40 mg/kg (p.o.) 3 weeks	↑ Sucrose intake	↑ 5-HT in HP and PFC	[40]
Hyperoside (quercetin 3-galactoside)	Male Albino Swiss mice	3.75 mg/kg (i.p.) 60 min before the behavioral test	↓ TTI in FST and TST	Pretreatment with pCPA (100 mg/kg, i.p.) prevented antidepressant-like effect of hyperoside	[176]
Quercetin	Male Swiss Albino mice	25 mg/kg (p.o.) for 4 weeks	↓ TTI in FST and TST	↑ Brain levels of 5-HT	[100]
Fisetin	Male ICR mice	10 and 20 mg/kg (p.o.) 60 min before behavioral test	↓ TTI in FST and TST	↑ 5-HT in frontal cortex and HP	[166]
Vixetin (apigenin-8-C-glucopyranoside)	Adult male BALB/c mice	10, 20, and 30 mg/kg (p.o.) 60 min before behavioral test	↓ TTI in FST and TST	Pretreatment with NAN 190, a 5-HT_1A_ antagonist (0.5 mg/kg, i.p.) prevented antidepressant-like effect of vixetin	[177]
Apigenin	Male ICR mice	7, 10, 14, and 20 mg/kg (p.o.) for 2 weeks	↓ TTI in FST ↑ Sucrose intake	↑ 5-HT in PFC, HP, hypothalamus and nucleus accumbens of rats exposed to CMS	[178]
	Albino mice (either sex)	25 and 50 mg/kg (p.o.) 24, 5, and 1 h before the behavioral test	↓ TTI in TST and FST	Pretreatment with pCPA (100 mg/kg, i.p.) prevented antidepressant-like effect of apigenin	[179]
Naringenin (4′,5,7-trihydroxyflavanone-7-rhamnoglucoside)	Male ICR mice	10, 20, and 50 mg/kg (p.o.) 60 min before the behavioral test	↓ TTI in TST	Pretreatment with pCPA (100 mg/kg, i.p.) prevented antidepressant-like effect of naringenin	[180]
	Three months old BALB/c male mice	25, 50, and 100 mg/kg (p.o.) for 14 days	↑ Sucrose intake ↓ TTI in FST	↑ 5-HT in cortex and HP	[181]
Silibinin	6–8 weeks old Kunming mice	100, 200, and 400 mg/kg (p.o.) for 3 weeks	↓ TTI in TST and FST	↑ 5-HT in PFC and HP	[182]
	Eight weeks old male Sprague Dawley rats	25, 50, and 100 mg/kg (i.p.) for 14 days	↓ TTI in the FST ↑ Sucrose intake	↑ 5-HT in HP and amygdala, and enhanced expression of TpH-1 mRNA in HP	[183]
Chrysin	Male C57B/6J mice	5 and 20 mg/kg (p.o.) for 14 days	↓ TTI in FST	↑ 5-HT in HP	[130]
	Adult female C57BL/6 mice	20 mg/kg (p.o.) for 28 days	↓ TTI in FST and TST	↑ 5-HT in PFC and HP	[184]
	Male Wistar rats	5 mg/kg (p.o.) for 28 days	↓ TTI in FST	↓ 5-HT_1A_ and 5-HT_2A_ mRNA in raphe nucleus ↑ 5-HT_1A_ mRNA in HP	[185]
Nobiletin	Male ICR mice	25, 50, and 100 mg/kg (p.o.) 60 min before the behavioral test	↓ TTI in FST and TST	Pretreatment with WAY 100,635 (7.1 mg/kg, s.c., a serotonin 5-HT_1A_ receptor antagonist) and cyproheptadine (3 mg/kg, i.p., a serotonin 5-HT_2_ receptor antagonist) prevented antidepressant-like effect of Nobiletin	[186]
Liquiritin (7-Hydroxyflavanone 4′-O-glucoside) and Isoliquiritin (2′,4,4′-Trihydroxychalcone 4-glucoside)	Mice	10, 20, and 40 mg/kg (p.o.) 30 min before the behavioral test	↓ TTI in FST and TST	↑ 5-HT in HP, hypothalamus and cortex	[187]

i.p. = intraperitoneally; p.o. = per oral rout; ↑ = the variable was increased; ↓ = the variable was decreased; TTI = total time of immobility; 5-HT = serotonin; HP = hippocampus; PFC = prefrontal cortex; FST = forced swim test; TST = tail suspension test; pCPA = p-chlorophenylalanine methyl ester; CMS = chronic mild stress; 5-HT_1A_ = 5-hydroxytryptamine 1A receptor; 5-HT_2A_ = 5-hydroxytryptamine 2A receptor CUMS = chronic unpredictable mild stress.

Concerning this, hesperidin, a flavonoid abundant in highly consumed citrus fruits such as oranges and lemons, is capable of crossing the BBB [188] and produces anti-inflammatory, antioxidant, and neuroprotective effects [188,189]. It has also been reported to produce antidepressant-like effects in murine models, e.g., the acute or chronic administration of 1 mg/kg hesperidin to mice or chronic administration of 20, 50, and 100 mg/kg hesperidin to rats reduced the immobility time in FST and TST [169,171] and increased sucrose intake [160] and 5-HT concentrations in the HP, PFC, and amygdala [171,172], while the pretreatment with p-chlorophenylalanine methyl ester (pCPA), a selective inhibitor of tryptophan hydroxylase, an important enzyme in the biosynthesis of serotonin, prevents the antidepressant-like effect of hesperidin [169].

Similarly, in preclinical research on mice, the acute administration of 10 and 20 mg/kg fisetin [166], a flavonoid found in fruits such as apples and strawberries, or acute (10, 20, and 50 mg/kg) [180] or chronic (25, 50, and 100 mg/kg) [181] naringenin, the predominant flavonoid in grapefruit, produces antidepressant-like effects, which are associated with increased 5-HT in the frontal cortex and HP that are abolished through pretreatment with pCPA [167,180,181], which implicates the serotonergic system in its pharmacological and behavioral effects.

In complement, chronic administration of apigenin (7 and 50 mg/kg) [179,180] or (5 and 20 mg/kg) chrysin [129,184], both flavonoids from plants *Passiflora incarnata* and *Matricaria chamomilla*, also increased motivation behaviors—less immobility in FST and TST and higher consumption of sucrose—mediated by the serotonergic system, with higher concentrations of 5-HT in the PFC, HP, and nucleus accumbens, all effects that were prevented by pretreatment with pCPA or ondansetron, a serotonin 5-HT_3_ receptor antagonist [131,178,184], important effects if we consider that these brain structures are involved in the physiopathology of depression and are pharmacological targets of antidepressant drugs (i.e., SSRIs, tricyclics, and MAOIs).

The data show that flavonoids have antidepressant-like effects that are related to the modulation of the serotonergic system, similar to that observed with clinical antidepressant drugs, highlighting the potential utility of flavonoids to produce therapeutic effects in humans.

## 9. BDNF Implicated in the Antidepressant-like Effect of Flavonoids

Several flavonoids exert different mechanisms through which they can modulate the BDNF system and are therefore able contribute to their antidepressant-like effect [16,190,191]. Among these flavonoids are hesperidin, apigenin, astibilin, bacalein, chrysin, dihydromyricetin, hyperoside, icariin, 7,8-dihydroxyflavone, myricetin, naringenin, naringenin, orientin, and silibinin [17]. Table 3 summarizes the actions on BDNF related to the antidepressant-like effect of flavonoids.

**Table 3:** Role of BDNF in the antidepressant-like effect of flavonoids.

Flavonoid	Experimental Subjects and Condition	Dose and Treatment Duration	Behavioral Effects Related with Antidepressant Activity	Effect on BDNF	Reference
Baicalein	Male Sprague–Dawley rats exposed to restrain stress	10, 20, and 40 mg/kg (i.p.) for 14 days	↓ TTI in FST ↑ Sucrose intake	↑ BDNF levels in HP	[192]
	Male C57BL/6J mice with depressive-like behavior induced by rotetone	300 mg/kg (p.o.) for 4 weeks	↓ TTI in TST ↑ Sucrose intake	↑ BDNF levels in HP ↑ TrkB phosphorylation	[193]
Icariin	Male C57BL/6J mice exposed to social defeat	5 and 10 mg/kg (p.o.) for 28 days	↑ Interaction time	↑ BDNF mRNA in HP	[194]
Hesperidin	Male adult Swiss mice	0.3 and 1 mg/kg (i.p.) for 21 days	↓ TTI in FST	↑ BDNF levels in HP	[167]
Astibilin	Male C57BL/6J mice exposed to CUMS	10, 20, and 40 mg/kg (i.p.) for 21 days	↓ TTI in FST and TST ↑ Sucrose intake	↑ BDNF levels in frontal cortex	[168]
Naringenin	Male ICR mice exposed to CUMS	10 and 20 mg/kg (p.o.) for 21 days	↑ Sucrose intake	↑ BDNF and mRNA BDNF in HP	[195]
7,8-dihydroxiflavone	Male C57BL/6 mice submitted to CMS	10 and 20 mg/kg (i.p.) for 28 days	↑ Sucrose intake	↑ BDNF in HP and PFC Agonist to the TrkB receptor	[196]
Chrysin	Female C57BL/6J mice exposed to CUMS	5 and 20 mg/kg (p.o.) for 28 days	↑ Sucrose intake ↓ TTI in FST	↑ BDNF and NGF in HP and PFC	[130]
Orientin	Male Kunming mice	20 and 40 mg/kg (p.o.) for 21 days	↑ Sucrose intake ↓ TTI in FST and TST	↑ BDNF in HP and PFC	[40]
3,5,6,7,8,30,40-Heptame thoxyflavone	C57BL/6 mice	50 mg/kg (s.c.) for 25 days	↓ TTI in FST and TST	↑ BDNF in HP	[197]
Apigenin	Male ICR mice	20 and 40 mg/kg (p.o.) for 21 days	↑ Sucrose intake ↓ TTI in FST	↑ BDNF in HP	[198]
Fisetin	Male ICR mice	5 mg/kg (p.o.) for 21 days	↓ TTI in FST and TST	↑ Activation of TrkB receptor in HP	[199]
Silibinin	Male Sprague-Dawley rats with depression-like behavior induced by Aβ1-42 oligomers	50 and 100 mg/kg (p.o.) for 15 days	↓ TTI in FST and TST	↑ BDNF and TrkB receptor expression in HP	[200]
Quercetin	Female C57BL/6J ERα-KO mice	10 mg/kg (p.o.) for 10 weeks	↓ TTI in FST and TST	↑ BDNF and TrkB receptor expression	[201]

i.p. = intraperitoneally; FTS = forced swim test; ↑ = the variable was increased; ↓ = the variable was decreased; TTI = total time of immobility; BDNF = brain derived neurotrophic factor; p.o. = per oral rout; TrkB = Tropomyosin receptor kinase B; mRNA = messenger ribonucleic acid; HP = hippocampus; CUMS = chronic unpredictable mild stress; TST = tail suspension test; PFC = prefrontal cortex; CMS = chronic mild stress; NGF = nerve growth factor; s.c. = subcutaneous injection.

According to the above, one of these reported mechanisms is the one that suggests that flavonoids possess a neuroprotective action mediated by the increase in BDNF levels, since they prevent induction of the depressive-like behavior in rodents submitted to depression models (i.e., TST, FST) [167,199,201]. For example, chronic pretreatment with hesperidin (0.3 and 1 mg/kg, i.p., for 21 days) increases the levels of BDNF in HP, which is associated with the decrease in immobility in the FST [154]. It has also been observed that the daily administration of baicalein (10, 20, and 40 mg/kg; i.p.) prior to daily exposure to repeated restraint stress (2 h/day) for 14 days increases BDNF levels and decreases corticosterone concentrations in the HP, which is related to prevention of depressive-like behavior in FST [192]. 

Moreover, these polyphenolic compounds can increase the expression of BDNF in the brain. In this sense, the antidepressant-like effect of chronic treatment with different flavonoids such as icariin (5 and 10 mg/kg, p.o., for 28 days), naringenin (10 and 20 mg/kg, p.o., for 21 days), silibinin (50 and 100 mg/kg, p.o., for 15 days), and quercetin (10 mg/kg, p.o., for 10 weeks) were associated with increased levels of BDNF mRNA, particularly in brain structures such as HP and PFC [194,195,200,201].

On the other hand, some flavonoids, in addition to regulating BDNF, can also modulate its receptor TrkB [193,197,199,200,201]. For example, chronic treatment with fisetin (5 mg/kg, p.o., for 21 days) increases the TrkB receptor activation in the HP of ICR mice [199]; similarly, sibylinin (50 and 100 mg/kg, p.o., for 15 days) promotes increased expression of this receptor in the HP of male Sprague-Dawley rats [200], actions that were both associated with a decrease in immobility behavior in the FST and TST, which is considered an antidepressant-like effect. These results have significant implications considering that the activity of BDNF and its TrkB receptor can independently regulate the therapeutic effects of conventional antidepressants [88]. The above suggests that flavonoids could exert their therapeutic actions through an alternative mechanism than regulating BDNF levels.

Flavonoids, at preclinical level, can also reverse depressive behaviors by increasing BDNF levels, such as in the case of depressed patients administered antidepressants [153]. For example, astibilin (10, 20, and 40 mg/kg, i.p., for 21 days) reversed the anhedonic behavior in male C57BL/6J induced by chronic stress, which was related to the increase in BDNF levels in the frontal cortex [168]. This same effect has been reported for naringenin (10 and 20 mg/kg, p.o., for 21 days) [182], 7,8-dihydroxyflavone (10 and 20 mg/kg, i.p., for 28 days) [197], and chrysin (5 and 20 mg/kg, p.o., for 28 days) [130], which confirms the potential of flavonoids as possible molecules that could be used for the development of pharmacological prototypes for the treatment of depression.

## 10. Concluding Remarks

Depression is one of the main comorbidities in many chronic diseases and a healthcare challenge due to the overlapping of symptoms and lack of adherence to treatment [202]. Therefore, having pharmacological alternatives with a greater efficacy and range of safety is one of the objectives and the main priority in preclinical research. In this sense, flavonoids represent a new pharmacological proposal for the treatment of depressive symptoms. These compounds have a wide repertoire of biological proprieties such as antioxidant, anti-inflammatory, anti-histaminic, anxiolytic, and antidepressant properties [105,107,108]. This last property has gained great interest in the neuropharmacology field, due to flavonoids having been demonstrated to exert antidepressant-like effects in different animal models [165].

As reviewed, there is extensive evidence suggesting that the serotonergic system and BDNF are the two main pathways through which flavonoids exert their antidepressant action. The evidence collected in this review shows that most of the described flavonoids modulate the serotonergic system by increasing the 5-HT levels in specific brain structures implicated in mood regulation, such as the HP and PFC (see Table 2). However, there are few studies that evaluate the participation of other components of the serotonergic system (i.e., enzymes and receptors, among others), which could also explain the increase in 5-HT levels. Despite this, the literature suggests that 5-HT is modulated by changes in the expression of the enzyme TpH-1, which is responsible for converting tryptophan to 5-HT [181], as well as the decrease in the mRNA levels of 5HT_1A_ and 5-HT_2A_ receptors in the raphe nucleus, which could increase the 5-HT firing rate in postsynaptic areas [183]. In addition, it is noteworthy that the effects of flavonoids on 5-HT levels are similar to those produced by conventional antidepressant drugs: the increase in 5-HT has been detected even 30 min after the administration of flavonoids [169], and the mechanism of action requires elucidation. Likewise, the effect of flavonoids on 5-HT levels persists during chronic administration [100,175] and has been widely associated with the increase in trophic factors and neuroplasticity processes. 

**Figure 1:**
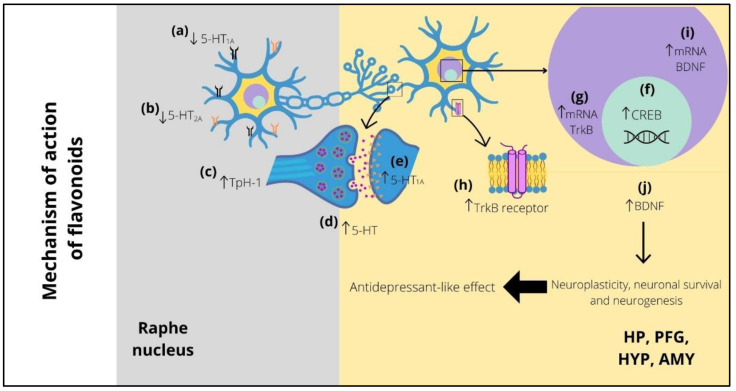
Possible implication of serotonergic system and BDNF in the antidepressant-like effect of flavonoids. Long-term treatment with flavonoids decreases the expression of presynaptic (raphe nucleus) 5-HT_1A_ (**a**) and 5-HT_2A_ (**b**) receptors and can also increase the expression of TpH-1 (**c**), both of which improve the 5-HT levels (**d**) in several postsynaptic areas (i.e., HP, PFC, HYP, and AMY), increasing the levels of postsynaptic 5-HT_1A_ receptors (**e**). The high levels of 5-HT promote CREB expression (**f**), which stimulates the increase in mRNA (**g**) and protein (**h**) of TrkB receptor. Similarly, the levels of mRNA (**i**) and protein (**j**) of BDNF are increased. The above improves the neuroplasticity, neuronal survival, and neurogenesis, which is reflected in the appearance of the antidepressant-like effect of flavonoids. 5-HT_1A_ = serotonin 1A receptor; 5-HT_2A_ = serotonin 2A receptor; Tph-1 = tryptophan hydroxylase 1; 5-HT = serotonin; HP = hippocampus; PFC = prefrontal cortex; HYP = hypothalamus; AMY = amygdala; CREB = cAMP response element-binding; mRNA = messenger ribonucleic acid; TrkB = tropomyosin receptor kinase B; BDNF = brain-derived neurotrophic factor.

With respect to the participation of BDNF in the antidepressant-like effect of flavonoids, this review shows that most flavonoids induce increased levels of this trophic factor, particularly in the HP and PFC (Figure 1). In addition, the onset of these effects has been reported after two weeks of treatment with flavonoids (see Table 3) similar to that of conventional antidepressant drugs. However, it is still necessary to explore pharmacological strategies that include the administration of flavonoids in combination with conventional antidepressants, which could reduce the latency of therapeutic effects, and to evaluate the possible participation of BDNF in the rapid facilitation of such effects.

The analyzed evidence also suggests that flavonoids can even promote TrkB pathway signaling through increasing the expression and activation of TrkB. This is relevant since these effects were also observed in studies where flavonoids (baicalein and quercetin) were administered orally, which would suggest that even their metabolism does not necessarily impose limitations with respect to exertion of their biological actions [193,200]. This could be useful on the design of pharmacological prototypes based on the structure of flavonoids with the aim of exerting rapid antidepressant effects, since the use of TrkB modulators has been shown to shorten latency to the appearance of therapeutic effects [87,203]. 

Additionally, despite describing the antidepressant-like effect of various flavonoids in this review, there is little literature evidence regarding the specific groups or features in their chemical structure that determine their antidepressant potential. However, in a recent study conducted in 2016, it was shown that those flavonoids with multiple hydroxy groups in their structure, such as apigenin (4′, 5, 7-trihydroxyflavone), quercetin (3, 3′, 4′, 5, 7-five hydroxyflavone), fisetin (3, 3′, 4′, 7-tetrahydroxyflavone), and luteolin (3′, 4′, 5, 7-tetrahydroxyflavone) have mostly been reported to have antidepressant properties. Thus, authors subjected these four flavonoids to a structure–activity relationship study and demonstrated that the presence of hydroxy groups in position five or seven on the A-ring is a common feature of these flavonoids. Another of the structural characteristics is the presence of C-glycoside, for example, in flavonoids such as icariin, rutin, and vixetin, in which either monosaccharides or disaccharides are present [99]. This information could imply an important guideline for the development of future molecules derived from flavonoids, which may have specific characteristics in their structure, which ensure an antidepressant profile.

Another point to consider is that, although most of the studies described in this review used oral administration, even then, antidepressant effects were observed; other research has also explored the effects of subcutaneous [196] and intraperitoneally injections [167,168,169,176,201], see Table 2 and Table 3. That could be seen as a limitation of flavonoids; however, these studies have been valuable to understand their potential therapeutic properties. Preclinical experiments exploring the effects of ketamine in animals commonly used intraperitoneal administration [204,205] in rodents and intravenous in primates [206], even though antidepressants are typically orally administered. These studies were valuable even though ketamine is currently administered by nasal spray [207]—for a review, see [208]—which has been approved for use in treatment-resistant depression in both the United States and Europe. Similarly, experiments studying flavonoids have been useful, especially when considering pharmacodynamic parameters in different routes of administration and would be valuable in the development of new therapies.

Focusing on another aspect, it is important to mention that most of the research described in this review is based on male individuals. Contrary to this, in humans, it has demonstrated that depression is more prevalent in women than men [209]. In addition, it is known that steroid hormones (i.e., estrogen, progesterone) play a fundamental role in the response to antidepressant drugs, both preclinically and in humans [210]. Therefore, it is still necessary to explore the interaction of flavonoids with steroid hormones regarding the modulation of serotonergic and BDNF systems on the antidepressant-like effects. Similarly, evaluating the possible side effects of flavonoids on sleep and sexual activity, among other behaviors, should be a goal to improve understanding on the pharmacological properties of theses polyphenolic compounds, considering that most of the studies on the antidepressant effects of flavonoids have only explored motor effects.

Finally, it has been shown that the antidepressant-like effects of flavonoids are related to their modulation of the serotonergic and BDNF systems. This is important, considering that new molecules with these properties could be designed for use as new treatments for depression. According to the multifactorial origin of depression, it would be interesting to consider the possible synergism of the anti-inflammatory, antioxidant, and regulation of the brain–microbiota axis effects promoted by flavonoids, a possibility that has recently been explored in some studies [101,181,211]. All of the compiled evidence thus highlights the importance of flavonoids in the development of new treatments for depression, emphasizing the necessity to continue exploring their pharmacological properties on the CNS and identifying potential side effects in screening promising candidates.

## Figures and Tables

**Table 1:** Classification and main characteristic of flavonoids.

		Structure	Examples of Compounds	Food Sources
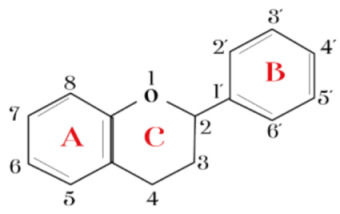 **Basic Skeleton**	Flavonol	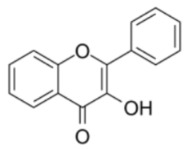	Quercetin, kaempferol, myricetin, and rutin	Olive oil, brocoli, apples, cherries, and berries
	Flavone	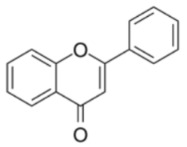	Chrysin, apigenin, luteolin	Red pepper, tomatoes, carrot, and chamomile
	Flavanone	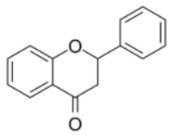	Naringenin, pinocembrin, and naringin	Orange, lemon, citrus, and grapefruit juice
	Flavanol	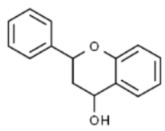	Catechin, epicatechin, epigallocatechin, and proanthocyanin	Grapefruit, lemon, apple, red wine, nuts, and tea
	Isoflavone	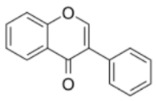	Genistein, Daidzein, and biochanin A	Soybeans and legumes
	Chalcone	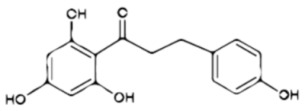	Naringenin-chalcone and pinocembrin-chalcone	Soy, apple, citrus, and ginger
	Anthocyanidin	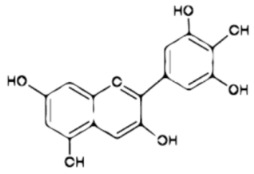	Cyanidin, delphinidin, pelargonidin, malvidin, and petunidin	Blackberries, cherries, strawberries, and chagalapoli

A and B = phenyl rings; C = heterocyclic ring.
